# Magnetoelectric and magnetodielectric coupling and microwave resonator characteristics of Ba_0.5_Sr_0.5_Nb_2_O_6_/CoCr_0.4_Fe_1.6_O_4_ multiferroic composite

**DOI:** 10.1038/s41598-018-30132-2

**Published:** 2018-08-02

**Authors:** Shivangi Tiwari, Satish Vitta

**Affiliations:** Department of Metallurgical Engineering and Materials Science, Indian Institute of Technology Bombay, Mumbai, 400076 India

## Abstract

A multiferroic composite consisting of single phases of 30 vol.% magnetostrictive ferrite and 70 vol.% relaxor ferroelectric has been synthesized. The ferrite exhibits a diffuse dielectric phase transition (DPT)with the transition temperature varying from 450 K to 600 K and an activation energy of 0.29 eV. Magnetically, it has a soft behavior with 70 emug^−1^ saturation magnetization and a Curie transition at ~620 K. The relaxor ferroelectric phase on the other hand exhibits two clear DPTs at 390 K–400 K and 150 K–300 K. The composite of these two shows a soft ferromagnetic behavior reminiscent of the ferrite along with 3 DPTs. There is strong coupling between the two orders – magnetostrictive and piezoelectric in the composite. The capacitance decreases by 45% in the presence of magnetic field corresponding to a sensitivity of 0.9% kOe^−1^, an extremely large value. The magnetoelectric coupling constant is found to be 20.6 mVcm^−1^Oe^−1^, a large value for a bulk composite. Microwave band stop filters of different thicknesses made from the composite have resonant frequencies which upshift in the presence of magnetic field indicating a multiferroic behavior with possibility for electric field tuning of resonant frequency.

## Introduction

The usefulness or value of a material increases manifold if it exhibits multiple interlinked functionalities. One such class of materials is ‘multiferroic materials’ wherein magnetic, electric and mechanical responses are interlinked. A magnetoelectric material for e.g. exhibits a change in electric polarization P on exposure to a magnetic field H and conversely a change in magnetization M in the presence of an electric field E. Such materials enable design and development of novel electronic devices for applications such as magnetic field sensors, energy harvesters, four state memories, and microwave filters etc^1–5^. In the case of microwave filters based on insulating magnetic ferrites, the tuning of resonant frequency can be achieved by varying the magnetic field. The magnetic fields required however are typically in kOe which make these devices not only bulky but also high power consuming^6,7^. Replacement of these ferrites with multiferroic materials facilitates electric field tuning of resonance frequency which makes them energy efficient as well as light. Since single phase materials that exhibit interlinked properties with high coupling are extremely difficult to find, composite materials that exhibit ‘multiferroic’ behaviour have become prominent. The individual components in such composites do not exhibit multiferroic behaviour but when in contact with each other, the two ferroic order parameters are coupled via a third parameter and the composite exhibits a magnetoelectric behavior. In the case of magneto electric composites the magnetic and electric orders are coupled via strain. The electric polarization P and the magnetization M of such a composite can be written as;

1 $$P={\delta }^{e,c}\sigma +{x}^{e}E+{\alpha }^{m}H$$

2 $$M={\delta }^{m,c}\sigma +{\alpha }^{e}E+{x}^{m}H$$

where δ^e/m^ and δ^e/m,c^ are direct and converse piezo-electric and piezo-magnetic coefficients, α^e/m^ are magnetoelectric coupling constants, χ^e/m^ are dielectric/magnetic susceptibilities and σ is the stress due to strain. The strain mediated transfer of order between the two components, α^e,m^ hence is a strong function of the nature of contact and is therefore an extrinsic parameter that can be tuned or controlled. An efficient packing of the two components that can result in maximum strain transfer results in maximizing the coupling constant α. In order to enhance the coupling constant various connectivity schemes such as 0–3, 2–2, 1–3, etc. between the two components have been extensively investigated. The most popular among them is the 2–2 scheme wherein both components are in alternating layered format, bilayers. The main advantage of this scheme is that the overall magnetoelectric coupling can be enhanced by just increasing the total number of bilayers in the 2–2 scheme structure. The disadvantage of this scheme however is that it requires sophisticated thin film deposition techniques which are not versatile in nature to deposit a variety of materials. In the present work a bulk composite with the magnetic component uniformly distributed in the piezo-electric matrix has been chosen due to the following reasons;

- The magnetic component in general is electrically more conductive compared to the piezo-electric component and hence uniform distribution of this component along with avoiding percolating conduction through this component can enhance the coupling constant.
- A bulk composite would be simpler to synthesize and hence will facilitate scale-up for applications as it does not use specialized processing procedures.

Several bulk magneto-electric composites with ferrites as the magnetostrictive component and either Pb-based or non-Pb-based oxides as piezo-electric component have been investigated to determine the coupling constant as a function of both the contents of the two components as well as processing parameters^8–14^. Some of the most recent results on bulk composites which are Pb-free are summarized in Table 1^15–25^. It is seen that preparation methods such as sol-gel in general lead to higher coupling constant. The objective of the present work however is to investigate the magnetoelectric coupling in a Pb-free relaxor/ferromagnetic-relaxor composite prepared by simple solid state method. Most importantly the use of this bulk magnetoelectric composite as a tuneable microwave filter has also been investigated. The ferroelectric relaxor component chosen is Ba_0.5_Sr_0.5_Nb_2_O_6_ while the magnetostrictive component is CoCr_0.4_Fe_1.6_O_4_. The ferroelectric relaxor Ba_0.5_Sr_0.5_Nb_2_O_6_ has been chosen due to its Pb-free nature and high piezo- and pyro-electric coefficients of 90 pCN^−1^ and 10^−8^ Ccm^−2^K^−1^ ^26–28^. It has a unfilled tungsten bronze crystal structure P4bm with a Curie transition temperature >300 K^29^. The magnetostrictive phase CoCr_0.4_Fe_1.6_O_4_ has been selected as the other component in the magnetoelectric composite due to its high strain rate derivative and Curie temperature 2 × 10^−9^ A^−1^m and 623 K respectively^30^. Partial substitution of Fe^3+^ with Cr^3+^ results in decreasing not only Fe^3+^ occupying the octahedral sites but also Co^2+^ in the cubic spinel structure. The resulting decrease in exchange interactions decreases the anisotropy constant and increases the strain rate derivative in this compound. It is found that the dielectric properties of this compound have not been studied well. Since its dielectric behaviour has an effect on the composite properties, the variation of dielectric constant and electrical conductivity of CoCr_0.4_Fe_1.6_O_4_ have also been studied in detail and discussed here. The magnetoelectric properties of composites of these oxides have been studied earlier as a function of varying content of the two components. It was found that composites with 0.3 mole fraction of CoCr_0.4_Fe_1.6_O_4_ distributed in Ba_0.5_Sr_0.5_Nb_2_O_6_ matrix exhibited a maximum magnetodielectric response of ~3.25% in a field of 8 kOe and 1 kHz electric field frequency at room temperature^31^. The variation of magnetodielectric response and magnetocapacitance of this composite as a function of electric field frequency, magnetic field and temperature however have not been investigated in detail. Hence this particular composite has been chosen for the present work and all the properties of this composite have been extensively investigated. Additionally, the microwave resonator characteristics of this composite have also been studied to explore application potential of this composite.

**Table 1:** The most recent data showing the magnetoelectric coupling constant α of various Pb-free multiferroic composites synthesized by different techniques.

Composites	Preparation Method	Frequency kHz	ME coefficient, α mVcm^−1^Oe^−1^	Ref.
0.8(0.6BaTiO_3_−0.4BiFeO_3_)−0.2(CoFe_2_O_4_)	Solid State Reaction	5	8.47	^15^
0.75(Bi_0.5_Na_0.5_TiO_3_)−0.25(La_0.67_Sr_0.33_MnO_3_)	Sol-gel method	1	19.1	^16^
80(BaTiO_3_)−20(CoFe_2_O_4_)	Solid State Reaction	1	1.29	^17^
0.9(K_0.5_Na_0.5_NbO_3_)–0.1(CoMn_0.2_Fe_1.8_O_4_)	Solid State Reaction	1	5.941	^18^
0.8[2.5(Bi_1/2_Na_1/2_TiO_3_)−22.5(Bi_1/2_K_1/2_TiO_3_)−5(BiMg_1/2_Ti_1/2_O_3_)]−0.2(NiFe_2_O_4_)	Solid State Reaction	1	73	^19^
0.7(Bi_0.5_Na_0.5_TiO_3_-Bi_0.5_K_0.5_TiO_3_)−0.3(Ni_0.8_Zn_0.2_)Fe_2_O_4_	Sol-gel method	1	126.5	^20^
80(0.5BZT-0.5BCT) – 20(NiFe_2_O_4_)	Sol-gel method	1	13.3	^21^
0.5[(Ba_0.85_Ca_0.15_)(Zr_0.1_Ti_0.9_)O_3_] – 0.5(CoFe_2_O_4_)	Sol-gel and auto-combustion	1	7.5	^22^
(0.94Na_0.5_Bi_0.5_TiO_3_−0.06BaTiO_3_)−0.2(CoFe_2_O_4_)	Solid State reaction	1	9.2	^23^
0.9[0.97(Bi_0.5_Na_0.5_TiO_3_)–0.03(K_0.47_Na_0.47_Li_0.06_Nb_0.74_Sb_0.06_Ta_0.2_O_3_)]− 0.1(Co_0.6_Zn_0.4_Fe_1.7_Mn_0.3_O_4_)	Solid State reaction	1.03	74	^24^
0.65(80Bi_0.5_Na_0.5_TiO_3_−20Bi_0.5_K_0.5_TiO_3_)−0.35(Ni_0.8_Zn_0.2_Fe_2_O_4_)	Sol -gel	1	42.41	^25^

## Experimental Methods

Solid state reaction technique was used to synthesize the two components of the composite, Ba_0.5_Sr_0.5_Nb_2_O_6_(SBN50) and CoCr_0.4_Fe_1.6_O_4_ (CCFO)^31^. Stoichiometric amounts of SrCO_3_, BaCO_3_ and Nb_2_O_5_ were mixed and Calcined at 1300 °C for 12 hrs. to synthesize the compound Ba_0.5_Sr_0.5_Nb_2_O_6_. To synthesize the ferrite stoichiometric amounts of Co_3_O_4_, Fe_2_O_3_ and Cr_2_O_3_ were mixed and Calcined at 1200 °C for 12 hrs. The compounds formation was verified by powder x-ray diffraction using Cu-K_α_radiation and subsequent Rietveld refinement of the diffraction spectrum using FullProf software. These two single phase compounds were then mixed together in the ratio (Ba_0.5_Sr_0.5_Nb_2_O_6_)_0.7_/(CoCr_0.4_Fe_1.6_O_4_)_0.3_ and pressed into a pellet to form the composite. The pressed pellets were then sintered at 1200 °C for 6 hrs. to form a dense composite pellet. The phase purity in the composite was also determined by a combination of powder x-ray diffraction and Rietveld refinement. The morphology of the grains was studied both in secondary and back scattered electron imaging modes in a scanning electron microscope to delineate the two phases clearly. The variation of magnetization M of the composite both with temperature T and external magnetic field H was investigated using a SQUID Magnetometer up to 800 K and ± 2 T fields. The variation of dielectric constant with T and frequency f was measured in the range 300 K to 600 K and 1 kHz to 100 kHz using a Novocontrol alpha analyser. Magneto-capacitance of the composite was studied up to 350 K in the presence of 5 T magnetic fields and from 1 kHz to 100 kHz using the Physical Properties Measurement System and an Agilent E4980A LCR meter. The sample for measurement of dielectric constant in the Novocontrol alpha analyser was in the form of a circular disc of 10 mm diameter and 1 mm thickness while the magneto-capacitance was measured using a rectangular bar sample. In all the measurements silver paste applied on the two faces acts as electrodes. Magneto-electric coupling in the composite at room temperature is also measured at a range of frequencies varying from 1 kHz to 15 kHz in ac magnetic fields up to 4000 Oe. An ac magnetic field of 1 Oe amplitude with varying frequency was superposed on the dc magnetic field. For magneto-electric measurement, electrical poling was done prior to measurements in a field of 400 Vcm^−1^ applied across the pellet for 45 min and measurements were done after 4 hrs of poling. The density of pellets used for all the measurements was >95% of theoretical density.For the realization of band stop filter, cylindrical pellet of the composite and CCFO were placed over a micro strip transmission line made on RT Duroid substrate. This substrate has low dielectric loss for frequencies <10 GHz while for higher frequencies its loss increases and hence is not ideal. The filter transmission coefficient S_21_ was measured by connecting the ports of the substrate to the network vector analyzer. For applying magnetic field perpendicular to the plane of the pellet, a permanent magnet capable of producing 5000 Oe magnetic field was used. To study the effect of varying dimensions of the filter on the dielectric resonance frequency, composites of different thicknesses, 0.9 mm and 2.4 mm but constant diameter of 10 mm were used.

## Results and Discussion

The microstructure of the composite together with that of the two constituent phases, CCFO and SBN50 was studied in detail using a combination of x-ray diffraction, Rietveld refinement of the diffraction patterns to determine the crystal structure and scanning electron microscopy. It was found that CCFO and SBN50 were both single phase with no additional/impurity phases and the composite was a mixture of these two phases only. The CCFO magnetostrictive ferrite was found to have an inverse spinel cubic structure corresponding to the space group Fd $\bar{3}\,$m with a lattice parameter of 0.8384(2) nm. The SBN50 relaxor compound has a tetragonal tungsten bronze structure corresponding to P4bm space group with a = 1.2463(9) nm and c = 0.3948(8) nm as the two lattice parameters. The CCFO phase was found to be randomly distributed in SBN50 matrix, an ideal microstructure to realize a large magnetoelectric coupling in a bulk system. The microstructural results and the crystal structure of the two constituents are given in the Supporting Information.

### Magnetization

#### CCFO

**Figure 1:**
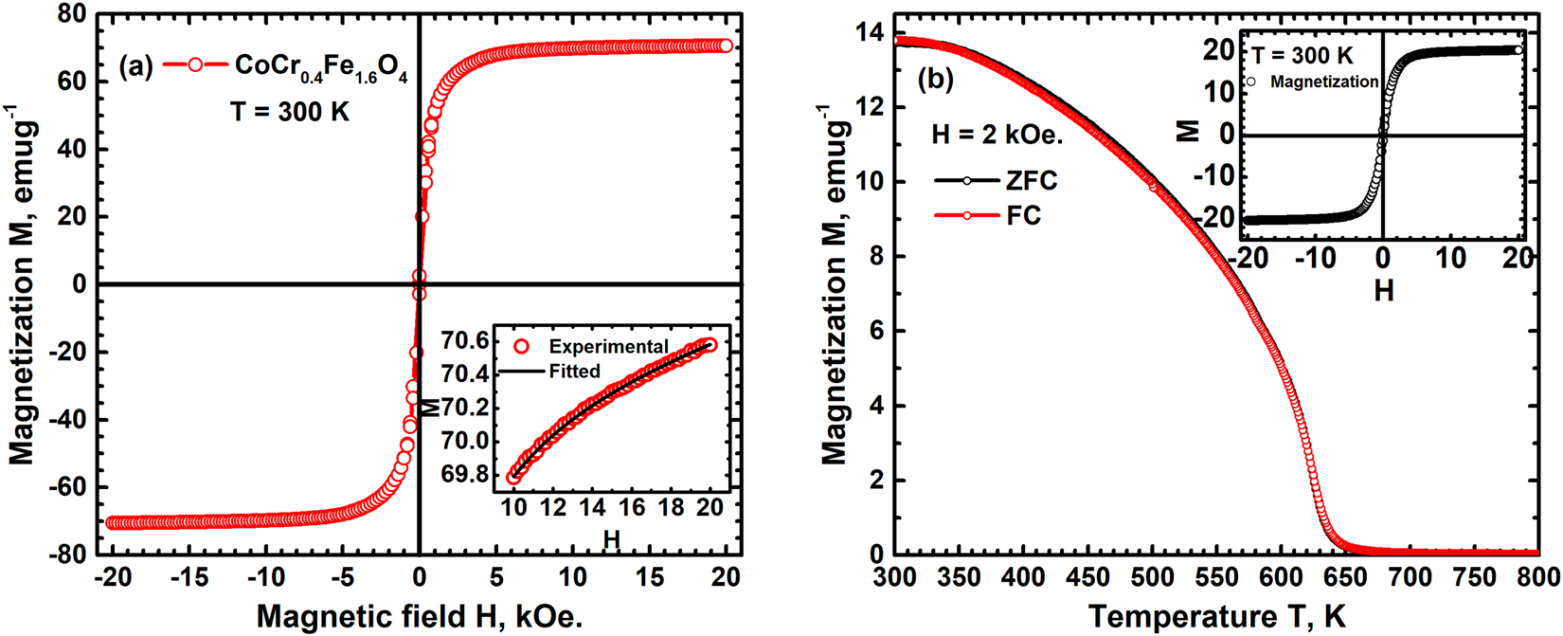
(**a**) The variation of magnetization M with external magnetic field H at room temperature of CCFO shows a soft ferromagnetic behaviour with low coercivity. The inset shows variation of M in the limit of H → ∞ fitting as per the rule of approach to saturation. (**b**) The high temperature variation of M of the composite in a field of 2 kOe shows thermal reversibility, zero-field cooled and field cooled. A clear magnetic transition at 620 K corresponding to Curie temperature of CCFO can be seen. The inset shows magnetic field hysteresis behavior of the composite at room temperature.

The variation of magnetization M with external magnetic field H at room temperature is shown in Fig. 1(a). The magnetization exhibits an extremely soft behaviour with a coercivity of ~30 Oe and near saturation with an M of 70 emug^−1^. The saturation magnetization of pure Co-ferrite has been reported to be ~80 emug^−1^ ^32^ and the lower saturation observed in CCFO is due to partial substitution of Fe^3+^ with Cr^3+^.The net magnetic moment μ_net_ of a ferrite in general therefore is a balance of magnetic moments of ions occupying tetrahedral and octahedral sites which have an antiferromagnetic interaction between them and is given by the relation;

3 $${{\rm{\mu }}}_{{\rm{net}}}=({{\rm{\mu }}}_{{\rm{oct}}}-{{\rm{\mu }}}_{{\rm{tet}}})$$

The cation occupancy between the two types of sites is determined by Rietveld refinement of the crystal structure and is given in Supplementary section Table 2. Using these occupancy factors for the cations the net magnetic moment of CCFO synthesized in the present work can be determined and is given by;

4 $${\mu }_{net}=[({\mu }_{C{o}^{2+}})0.8+({\mu }_{F{e}^{3+}})0.8+({\mu }_{C{r}^{3+}})0.4]-[({\mu }_{C{o}^{2+}})0.2+({\mu }_{F{e}^{3+}})0.8]$$

The net magnetic moment μ_net_ corresponding to this occupancy is found to be 3.0 μ_B_ in agreement with the experimentally determined value of 2.94 μ_B_. These results clearly show that CCFO has an inverse spinel structure with antiferromagnetic interactions. The room temperature first order magnetocrystalline anisotropy energy density K_1_ can be determined using the approach of M to saturation for fields H ≫ H_c_ the coercive field. The variation of M with H in this limit is given by the relation^32^;

5 $$M(H)={M}_{s}(1-\eta /{H}^{2})+kH$$

where η is a constant related to K_1_ and k a constant related to paramagnetic component. This second term for a ferromagnetic material will be extremely small and can be neglected. The magnetization M for H >10 kOe is fitted to this equation, Fig. 1(a) and the value of η is found to be 5.2 × 10^9^ A^2^m^−2^. For a polycrystalline cubic anisotropy material with random alignment of crystallites K_1_ = μ_0_M_s_[η(105/8)]^1/2^ and hence K_1_ determined using the value of η obtained by fitting is found to be 1.2 × 10^5^ Jm^−3^. This value is lower compared to that of unsubstituted CoFe_2_O_4_, ~2.5 × 10^5^ Jm^−3^,^33^ clearly showing the effect of substituting Cr^3+^ resulting in displacing Co^2+^ to tetrahedral sites and thus promoting antiferromagnetic interactions.

### SBN50/CCFO Composite

The variation of M with temperature T in zero field cooled and field cooled conditions in the presence of 2 kOe external field is shown in Fig. 1(b). The composite exhibits a magnetic transition at ~620 K corresponding to the Curie temperature of CCFO showing that the magnetostrictive component has not undergone any change in the composite. The magnetization in both zero field cooled and field cooled conditions is identical and reversible with no thermal hysteresis. This shows that domain wall motion and formation are independent of field and temperature change directionalities. The room temperature hysteresis of M of the composite is shown in inset of Fig. 1(b). It has an extremely soft magnetic behaviour with H_c_ of ~70 Oe and a saturation magnetization of ~20 emug^−1^ compared to 70 emug^−1^ for the pure magnetostrictive phase. The M_s_ value of the composite corresponds to ~0.29 fraction of CFO and indicates a near linear decrease with increasing fraction of SBN50. The fraction of CFO determined based on M_s_ is in complete agreement with that determined from x-ray diffraction results and starting mix of the two constituents.

### Dielectric behaviour of CCFO

**Figure 2:**
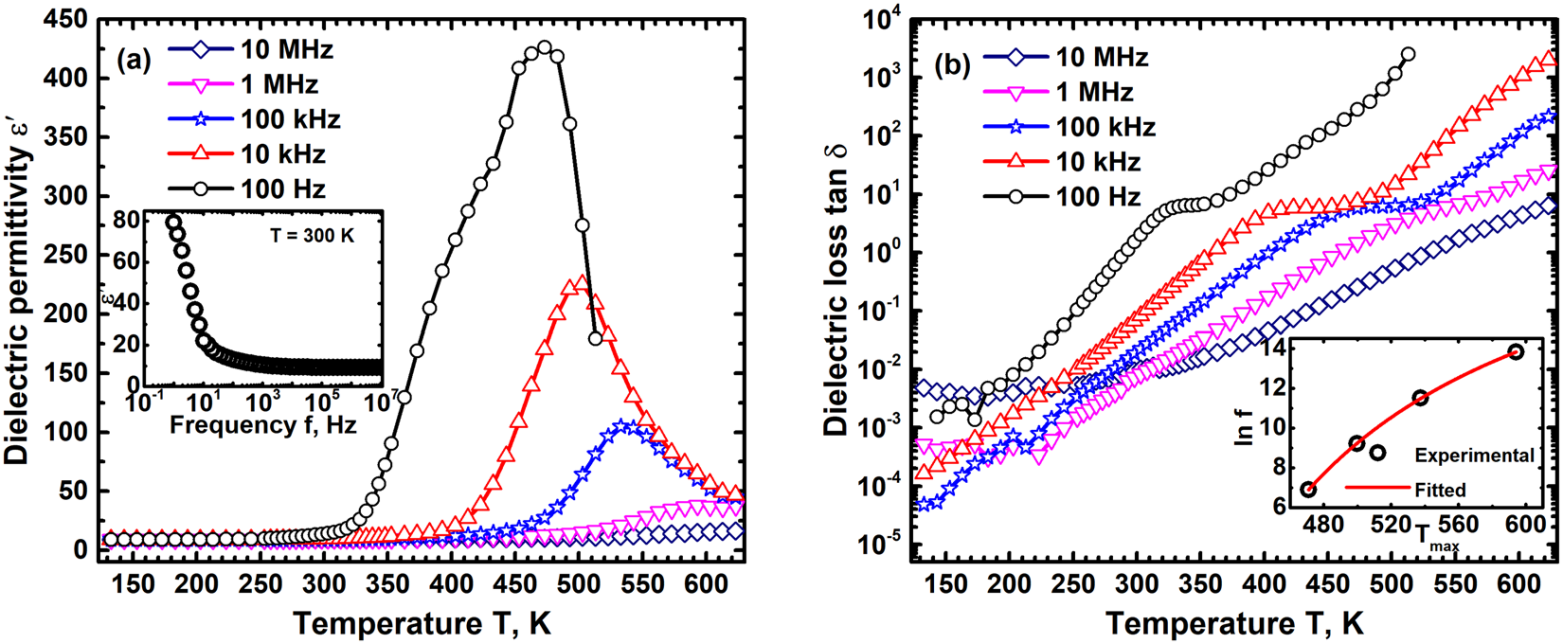
(**a**) The dielectric permittivity ε′ variation of CCFO with T at different frequencies f shows a diffuse phase transition, similar to relaxor ferroelectrics in the range 450 K to 600 K over 5 orders of magnitude variation in f. The inset shows a typical dielectric behavior at room temperature with ε′ decreasing with increasing f. (**b**) The dielectric loss tan δ also indicates a diffuse phase transition with the loss peak shifting to high temperature with increasing f. The permittivity peak temperature at different frequencies follows a Vogel-Fulcher behavior, inset, showing viscous relaxation.

The dielectric permittivity ε′ of CCFO decreases with increasing frequency in the range 1 Hz to 10 MHz at room temperature Fig. 2(a) inset, a behaviour commonly observed in ferroelectric materials. In the case of polycrystalline materials both interfacial (grain boundary) and hopping (within grains) polarizations contribute to the dielectric behaviour^34^. The grain boundary resistance in these materials in general will be higher than the bulk grain resistance. The limiting polarization therefore will be dominant due to the large grains present in the structure. The charge transport within the grains is due to hopping of carriers between multiple valence state ions and this process lags behind the frequency change of applied electric field. This lag decreases the effective dielectric permittivity at large frequencies. The hopping process however is thermally activated due to which the permittivity increases with increasing T as shown in Fig. 2(a). The permittivity ε′ exhibits a peak which shifts to higher temperatures with increasing frequency, dispersive behaviour typically found in ferroelectric relaxors. The peak at 100 Hz frequency has a dielectric permittivity of 420 at ~472 K and reduces to 100 at ~538 K in a field of frequency 100 kHz. The permittivity peak temperature increases by 125 K when the frequency increases by 4 orders of magnitude. This paraelectric to ferroelectric relaxor transition temperature range is close to the magnetic Curie temperature T_C_ of 620 K showing that magnetic phase transition is responsible for the ferroelectric relaxor behaviour. The large dispersion in peak temperature T_m_ indicates the weak ferroelectric nature and a relaxor like behaviour of this magnetostrictive oxide. The dielectric loss tan δ also exhibits dispersive peaks in this temperature range, Fig. 2(b). The dielectric loss however increases with T due to an inherent increase in electrical conductivity at high T. The high electrical conductivity of CCFO results in significant loss as seen in Fig. 2(b) which decreases with increasing frequency.

**Figure 3:**
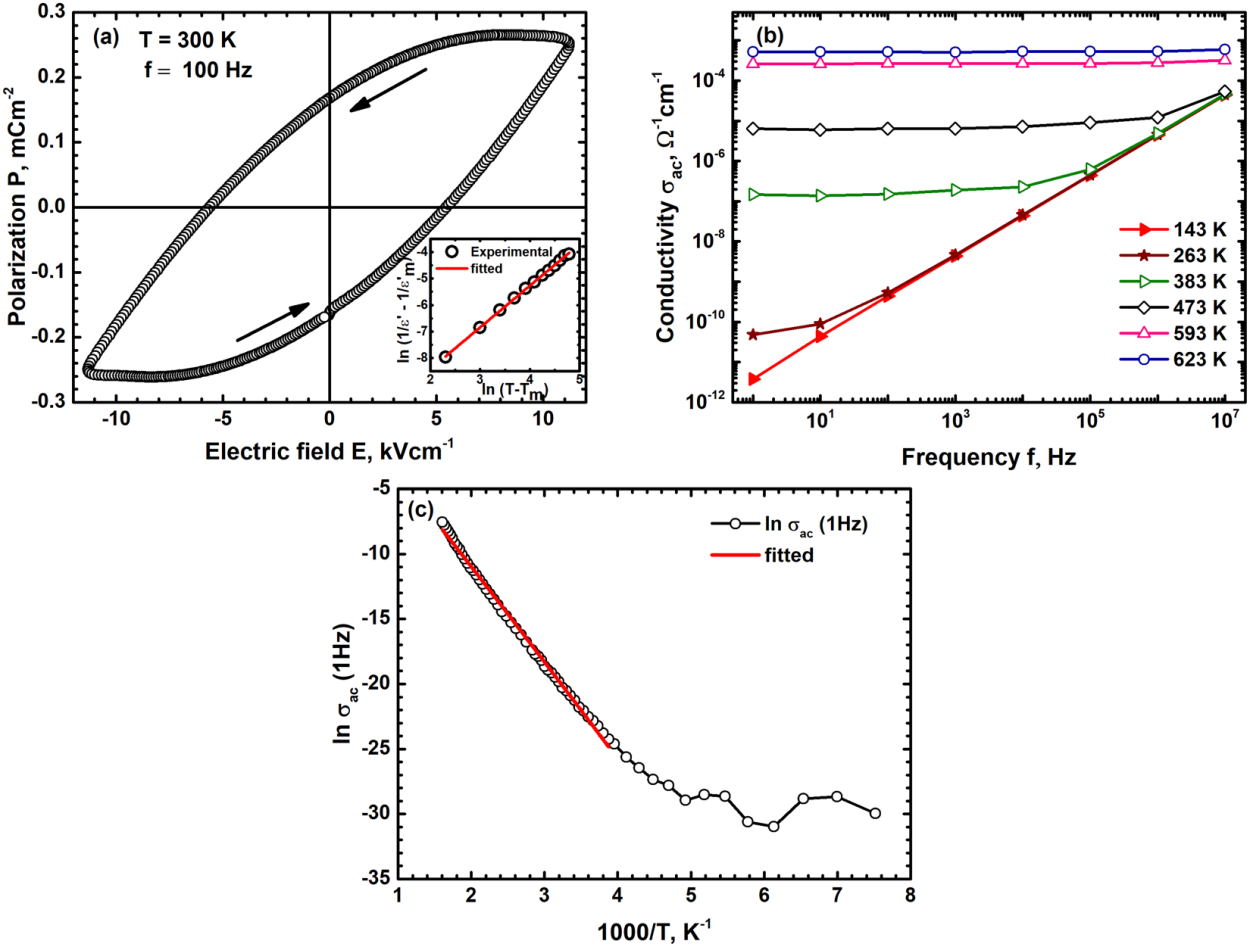
(**a**) The polarization P – electric field E loop at room temperature and 100 Hz frequency shows a ferroelectric nature with clear remanence and coercivity for CCFO. (**b**) The variation of electrical conductivity σ_ac_ at different T and f shows a typical dielectric behavior. The conductivity saturates at low frequency and increases with increasing frequency. The variation of σ_ac_ (1 Hz) with T shown in (**c**) exhibits a thermally activated small polaron temperature dependence.

The polar domains distributed in the relaxor state have a random orientation either in a ferroelectric or non-ferroelectric matrix. The orientation of these domains in an electric field is governed by their relaxation kinetics and it has been generally observed to follow a Vogel-Fulcher behaviour. Hence the frequency dependence of ε′ maximum temperature T_m_ has been fitted to the Vogel-Fulcher relation given by^35^;

6 $$f={f}_{0}exp(-{E}_{a}\,/\,{K}_{B}[{T}_{m}-{T}_{f}])$$

where ${f}_{0}$ is the ionic jump attempt frequency, E_a_ the activation energy for this jump and T_f_ the freezing temperature. The dielectric permittivity dispersion over 5 orders of magnitude has been fitted to the Vogel-Fulcher relation and the attempt frequency is found to be 10^10^ Hz with an activation energy of 0.29 eV and freezing temperature of 280 K. The activation energy, 0.29 eV is similar to the activation energy required for the oscillators’ relaxation and is due to hopping of charge carriers mediated by phonons. These results, viscous relaxation of ions, clearly show that below 280 K the polar regions are frozen for all frequencies and the oxide exhibits a net ferroelectric polarization below this temperature. The permittivity peak at 100 Hz is found to be at 472 K indicating that this compound should exhibit a ferroelectric behaviour at 300 K at 100 Hz. Hence the polarization P was measured as a function of electric field E at this frequency and the resulting P-E loop is shown in Fig. 3(a). It exhibits a clear ferroelectric behaviour with a remnant polarization of ~0.17 mCm^−2^ and a coercive field of ~5.6 kVcm^−1^. The temperature and frequency dependence of ε′ above T_m_ the permittivity peak temperature can be understood using the modified Curie-Weiss law given by^35^;

7 $$(1/\varepsilon ^{\prime} -1/{\varepsilon ^{\prime} }_{m})=\frac{{(T-{T}_{m})}^{\gamma }}{C}$$

where $\,{\varepsilon ^{\prime} }_{m}$ is the permittivity maximum at T_m_, C the Curie constant and γ the exponent which defines the nature of phase transition. The value of γ is 1 for a ferroelectric material which will not exhibit any dispersion and 2 for an ideal ferroelectric relaxor material. A fit of the permittivity at 10 kHz to the modified Curie-Weiss law, shown in inset of Fig. 3(a) gives γ of 1.57 clearly showing the relaxor like ferroelectric behaviour of CCFO. Recently ferroelectric properties were also observed in several centrosymmetric compounds in which the polarization develops due to the exchange of electronic charge between different cationic states or due to spin reordering^36–38^. The ac electrical conductivity σ_ac_ determined from dielectric constant as a function of T in the range 140 K to 630 K and 7 orders of variation in frequency f is shown in Fig. 3(b). It exhibits a classical dielectric behaviour with the conductivity varying significantly at high frequencies and becoming a constant at low frequencies. At low temperatures it has a significant ac component which decreases with increasing temperature. The total conductivity σ_ac_ increases with increasing temperature at all f. Hence the conductivity at 1 Hz is considered as dc conductivity σ_dc_ and its variation with temperature is shown in Fig. 3(c). The conductivity is thermally activated with a clear transition at ~260 K indicating a change in transport mechanism at this temperature. This temperature is nearly identical to the freezing temperature T_f_ of 280 K indicating that relaxor behaviour leads to changes in transport mechanism also. The low temperature conductivity is due to classical double exchange phenomenon between the different cations mediated by O^2−^ ions. The high temperature behaviour is found to follow a small polaron hopping behaviour with the conductivity varying with temperature as $\sigma (T)=({\sigma }_{0}/T)exp(-{E}_{p}/{k}_{B}T)\,$where σ_0_ is the limiting conductivity and E_p_ is the small polaron hopping energy^39^. A fit of σ_ac_(1 Hz) to this temperature dependence shown in Fig. 3(c) gives an activation energy of ~0.6 eV. The bulk conduction activation energy is found to be greater than the oscillators’ relaxation energy of 0.29 eV indicating a large band gap in these oxides^40^. These results clearly show that the high temperature conductivity is dominated by disorder with majority of the energy required for the formation of defects for ion movement. The rearrangement of Co^2+^, Fe^3+^, Fe^2+^ and Cr^3+^ ions between the tetrahedral and octahedral sites to form a ferromagnetic order also results in the development of local polarization which is frozen below 280 K.

### Dielectric behaviour of SBN50

**Figure 4:**
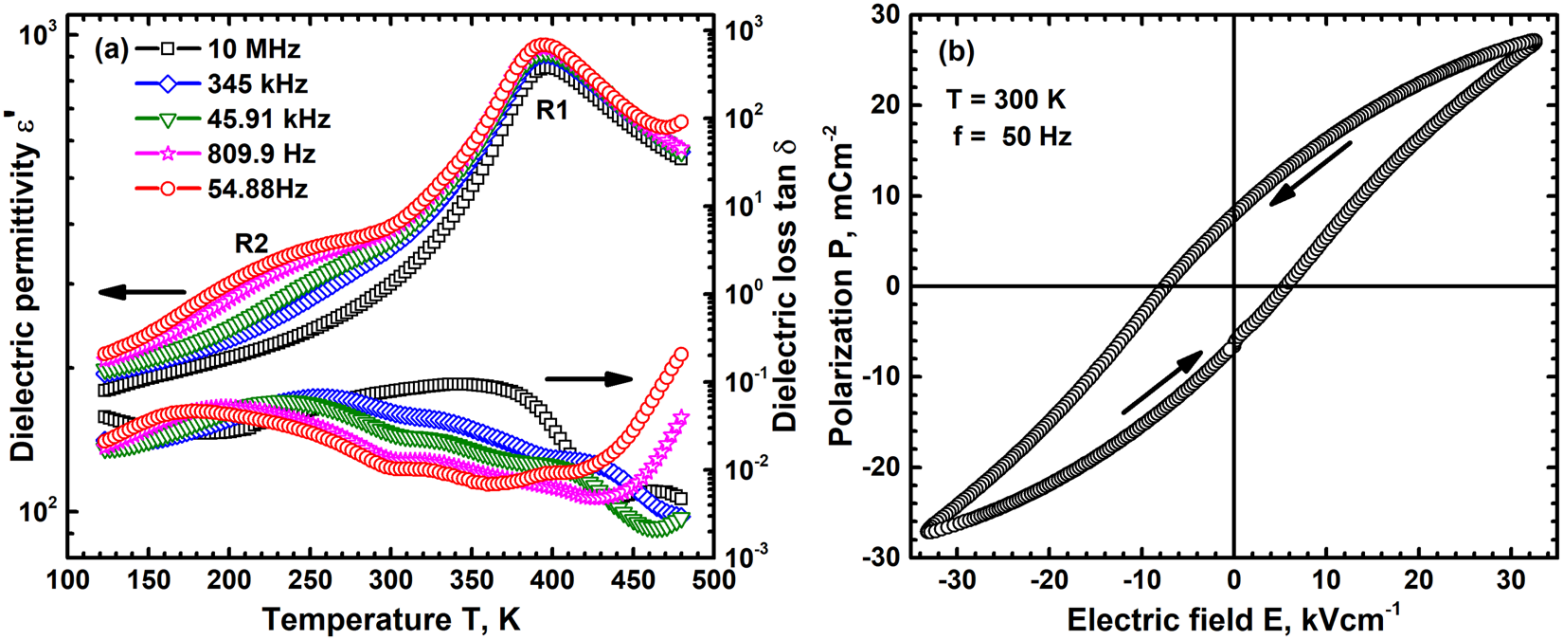
(**a**) The dielectric permittivity ε′ and loss tan δ variation of the relaxor ferroelectric SBN50 in the frequency range 50 Hz to 10 MHz shows two diffuse phase transitions R1 and R2 at different temperatures. (**b**) The polarization P variation with electric field E at room temperature and 50 Hz shows a typical ferroelectric behavior.

The dielectric permittivity ε′ and loss tan δ of SBN50 in the temperature range 100 K to 480 K and at frequencies varying from 50 Hz to 10 MHz is shown in Fig. 4(a).Two distinctly different relaxation features are observed in the permittivity curve which are denoted as R1 and R2 and both these are dispersive in nature. The transition temperatures in permittivity and loss however are not exactly coincident and such phenomena have been observed in this compound earlier. This is due to highly non-linear nature of dielectric and optical properties in these temperature ranges^41^. The high temperature relaxation R1 is in the temperature range 390 K to 400 K with the permittivity decreasing from ~960 to 870 as the frequency increases from 50 Hz to 10 MHz. This phenomenon is similar to the dispersive relaxation observed in ferroelectric single crystals of SBN50 and in the present case is close to the paraelectric-ferroelectric Curie transition temperature of 413 K observed for the SBN40 compound^29^. The magnitude of dispersion, ΔT_m_ however is very small 3 K for a frequency change of 6 orders of magnitude indicating the closeness to conventional ferroelectric behaviour. The peak temperature of dispersion therefore could not be fitted to either the simple Arrhenius or Vogel-Fulcher behaviours. The peak temperatures are sensitive to the nature of defects in this compound and these have been reported to vary from ~350 K to 375 K depending on the synthesis procedure adopted^29,42,43^. The smaller dispersion and higher T_m_ observed in the present work clearly show that the amount of Sr in the compound is lower than the nominal value of 0.5. Frequency dependent dispersive nature of ε’ in the temperature range from ~150 K to ~300 K i.e. R2 transition is visualized only at low frequencies. This peak corresponding to the R2 relaxation shifts to high temperatures with increase in frequency but vanishes for frequencies >10^4^ Hz.This low temperature dispersion behaviour follows a Arrhenius behaviour with an activation energy of ~0.43 eV indicative of ionic hopping. For high frequencies the permittivity increases continuously with increasing temperature till the R1 transition. Structurally, SBN50 is known to have several commensurate and incommensurate forms existing at room temperature which are ferroelectric and ferroelastic in nature. The room temperature phase is thus known to have intergrowth defects with a orthorhombic superstructure in a matrix of tetragonal tungsten bronze structure. A mixture of such forms leads to a chaotic state which exhibits relaxor like dielectric relaxation, R1 transition^41,43,44^. The nature of this transition and extent of dispersion therefore depends on fractions of the different forms present and a narrow temperature range of dispersion indicates that ferroelectric forms are dominant at this temperature. The room temperature polarization, between R1 and R2 transitions, shown in Fig. 4(b) exhibits a ferroelectric behaviour with a maximum polarization of ~30 mCm^−2^ and a coercive field of ~75 Vm^−1^ at 50 Hz. This P-E loop confirms the ferroelectric nature of SBN50 at room temperature. The lower temperature transition R2 is due to growth of commensurate structures or conversion of incommensurate structures which results in increasing the ferroelectric phase fraction. These dielectric and structural transitions can be understood within the context of recently proposed slush-like relaxation process in relaxor ferroelectric materials^45^. In analogy to this model, above the Burns temperature T_b_, the R1 transition, the materials has random polarization with net paraelectric behaviour. On cooling the system below T_b_ fluctuating domains of different size form with extremely small relaxation times. These fluctuating domains progressively grow and freeze with decreasing temperature, finally freezing to a state with extremely large relaxation times and a net ferroelectric behaviour at low temperatures, below R2. The low temperature diffuse phase transition R2 has been observed to be due to the formation of a incommensurate superlattice structure whose lattice parameter changes with decreasing temperature. This leads to a dispersive nature of the transition which can be seen only at low frequencies.

The dielectric relaxation of both CCFO and SBN50 exhibit typical relaxor like features and not classical ferroelectric behaviour. In typical ferroelectrics polarization is clearly earmarked between that of bulk grains and grain boundaries. In relaxors however the role of grains and grain boundaries is not clearly known as polarization in these materials is due to the formation of polar nano regions whose physical location in these materials microstructure can be anywhere. Also, these regions have short range order while in ferroelectric materials the order is long range in nature. Hence an attempt to analyse the dielectric relaxation spectrum in terms of an equivalent circuit model wherein the grains and grain boundaries are treated as independent entities may not be very correct for a relaxor ferroelectric material. The modelling of room temperature dielectric constant variation with frequency of CCFO however shows two clear relaxation entities, 1 and 2 in the microstructure. The fitting parameters obtained based on equivalent circuit model with a constant phase element model^46^ are R_1_ = 2.1 × 10^7^ Ω; R_2_ = 6.8 × 10^6^ Ω; R_3_ = 1.2 × 10^8^ Ω; C_1_ = 2.5 × 10^−9^ F; C_2_ = 4.3 × 10^−11^ F and C_3_ = 9.4 × 10^−12^ F. The hopping conductivity constant is found to be 0.65 indicating the dominance of bulk conduction. This model however does not work well to predict the dielectric behaviour of SBN50, indicating its limitation for relaxor ferroelectric materials.

### Dielectric behaviour of Composite

**Figure 5:**
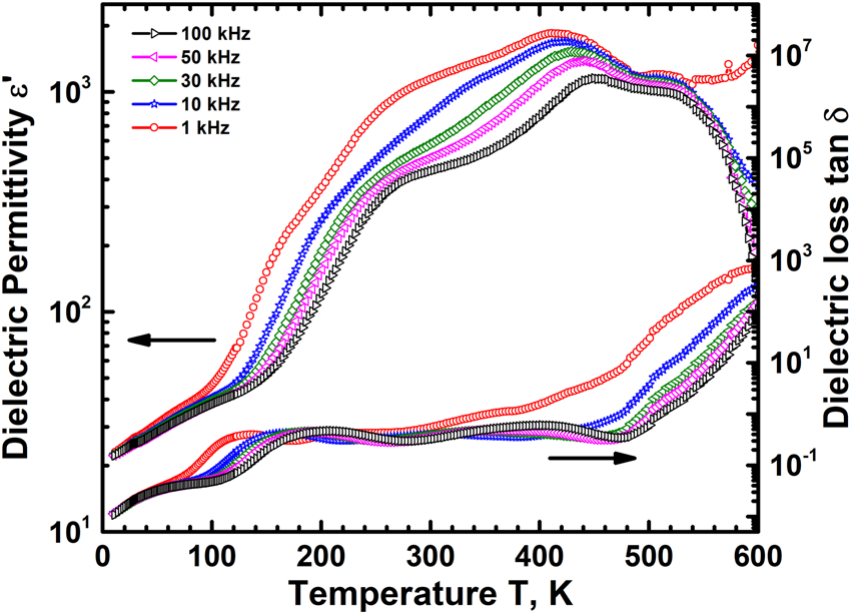
The dielectric permittivity ε′ and loss tan δ variation of the composite measured as a function of f and T shows 3 dispersive phase transitions at different temperatures. These transitions coincide with those of individual components, CCFO and SBN50 showing that the two components retain their individual behavior.

The dielectric permittivity ε′ and loss tan δ of the composite in the temperature range 10 K to 600 K and at frequencies varying from 1 KHz to 100 KHz was studied. It is found that for T <100 K the permittivity and loss are extremely small and nearly independent of frequency. Above 100 K however the composite exhibits a ferroelectric relaxor behaviour with the permittivity having several dispersions in the temperature range 100 K to 550 K, Fig. 5. The permittivity has three peaks – a broad low temperature peak below 250 K, a clear peak between 400 K and 450 K and third peak in the range 500 K to 550 K. All the three peaks shift to high temperatures with increasing frequency indicating that the three temperatures represent a relaxor like diffuse phase transition. The dielectric loss mirrors the dispersive nature of permittivity and is found to increase with increasing temperature at all frequencies due to conducting nature of CCFO component in the composite. These results clearly show that the composite has a ferroelectric relaxor behaviour with a diffuse phase transition which is a combination of that due to SBN50 and CCFO - the low and intermediate temperature transitions due to the SBN50 constituent while the high temperature transition is due to the CCFO component. The transition temperatures observed in the composite however do not match exactly with those obtained in the individual components due to macroscopic nature of ferroelectric transitions which depend strongly on the microstructure. In the case of composites the microstructural features such as nature of inter-phase grain boundaries, spatial distribution of the two phases, the fraction of each of the constituents and their grain sizes & distributions all have a strong effect on the transition characteristics. The presence of 3 distinct dielectric transitions in the composite which broadly coincide with those of the individual components however indicates that the ferroelectric and magnetostrictive phases in SBN50/CCFO composite retain their characteristic features. The high temperature relaxation corresponding to that of SBN50 follows a Vogel-Fulcher type behaviour with an activation energy of just 0.02 eV, in agreement with that of pure SBN50 which shows extremely small dispersion. The low temperature relaxation however is thermally activated with an activation energy of 0.15 eV. These results further confirm that the dielectric relaxation kinetics in the composite are governed by the microstructural features.

The equivalent circuit model to analyse the dielectric spectrum of a composite is non-trivial as the composite is made of electrically dissimilar components with large difference in grain sizes. The distribution of different components in the composite can have a significant effect on the total dielectric constant. Hence in the present work this model has not been used for the deconvolution of the role of grains and grain boundaries of the two electrically dissimilar components in the composite.

**Figure 6:**
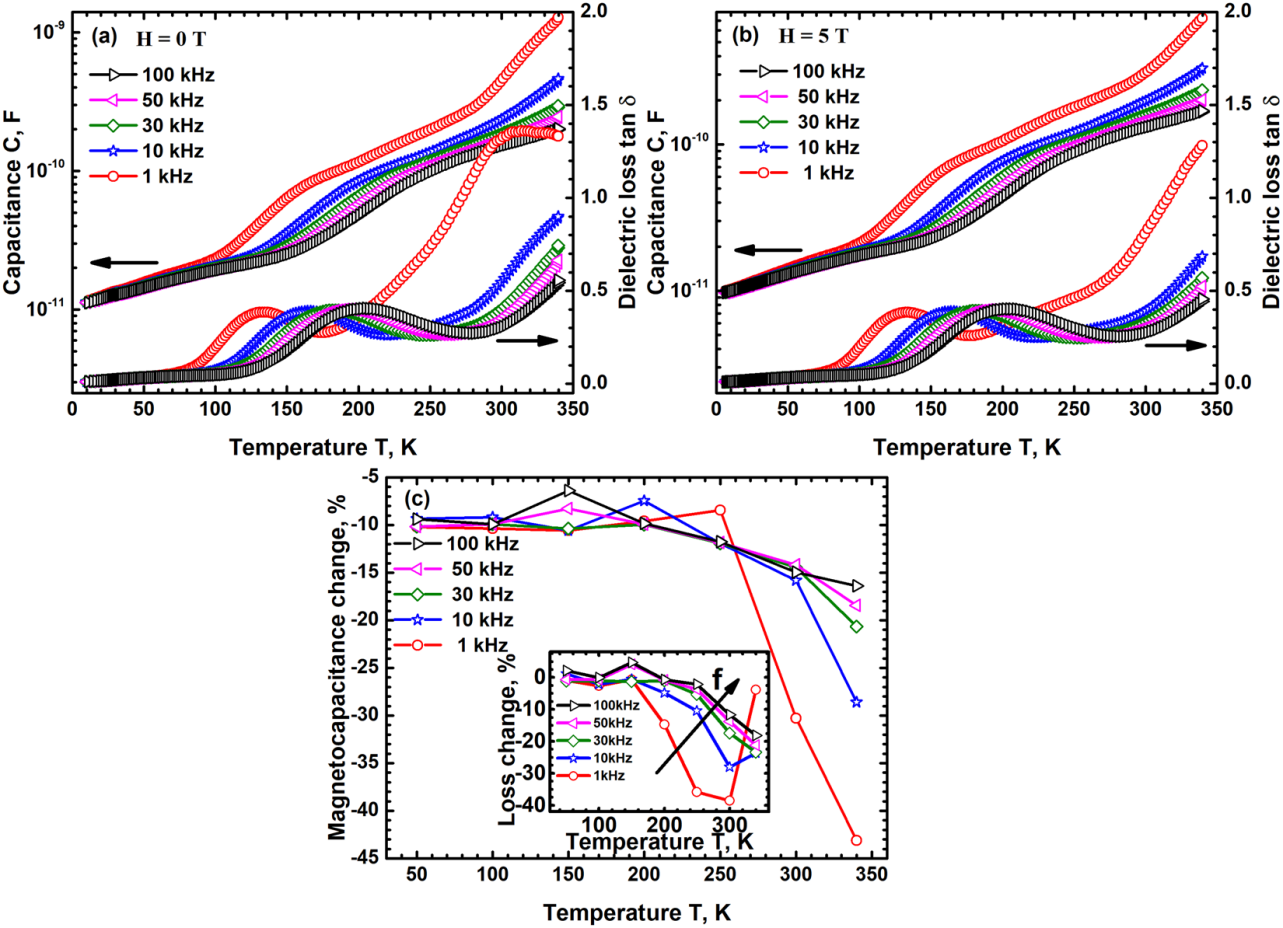
The change in capacitance and loss tan δ of the composite in the frequency range 1 kHz to 100 kHz and at temperatures up to 350 K in the absence (**a**) and presence of 5 T external magnetic field (**b**) are identical except for their absolute magnitudes. (**c**) The capacitance decreases in the presence of magnetic field and the magnitude of decrease increases with increasing temperature. The capacitance decrease however becomes smaller with increasing f.

The change in capacitance and hence the loss on application of an external magnetic field of 5 T to the composite capacitor in the temperature range 50 K to 340 K was studied and the results are shown in Fig. 6(a,b). The capacitance and loss in the presence of magnetic field follow an identical temperature and frequency dependence as those in the absence of magnetic field. The absolute magnitude however is lower in the presence of magnetic field and the difference increases with increasing T, Fig. 6(c). The decrease is found to be highest near room temperature clearly showing the applicability of the capacitor. The capacitance change is found to be ~45% near room temperature indicating a sensitivity of 0.9% kOe^−1^. The magnitude of capacitance decrease is found to be a function of frequency and decreases with increasing frequency to 16% at 10^5^Hz corresponding to a sensitivity of 0.32% kOe^−1^. These capacitance changes are large and shows the extent of magneto-dielectric coupling in the composite. This relatively large coupling shows that the magnetostrictive strain is effectively transferred to the relaxor ferroelectric and that the microstructure, volume fractions and the distribution of the two phases is optimal for 3-0 bulk composite.

### Magneto-electric coupling

**Figure 7:**
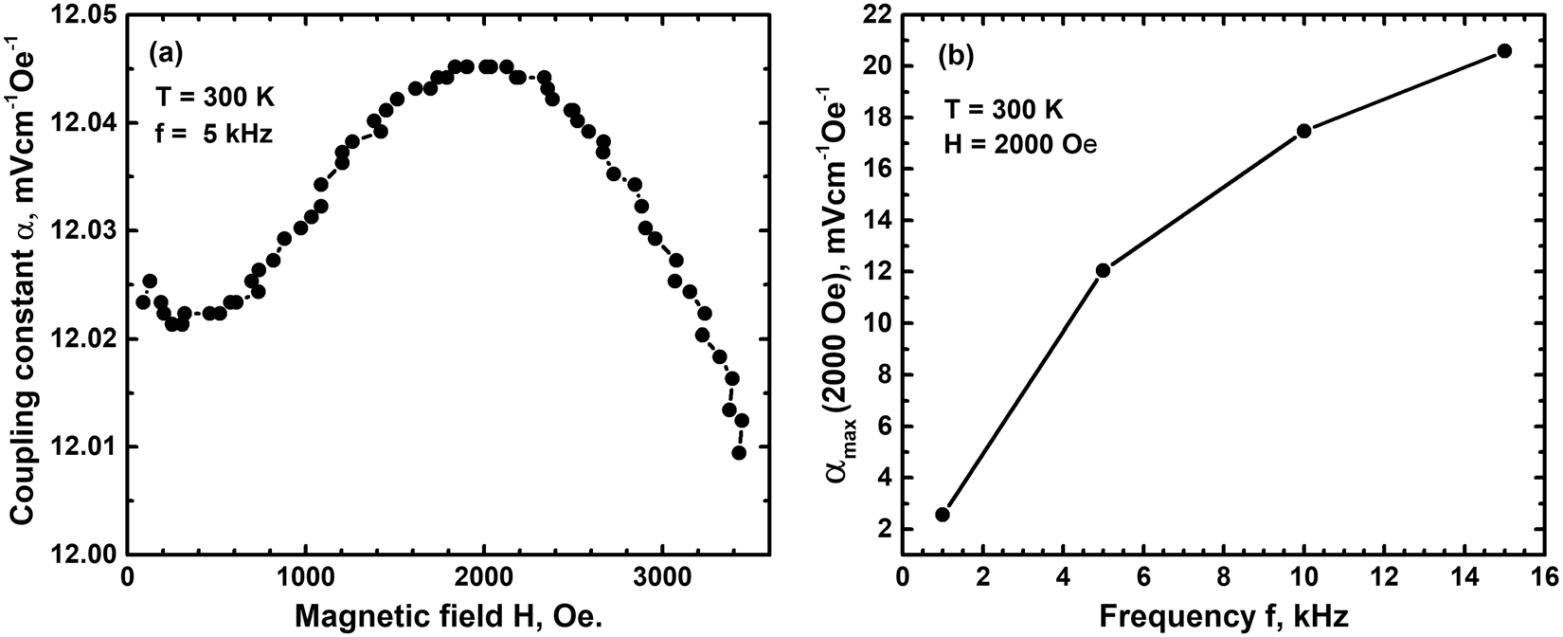
(**a**) The room temperature magneto-electric coupling constant α increases with increasing magnetic field H at all frequencies up to 2000 Oe external magnetic field and then decreases. (**b**) The maximum coupling constant α_max_ (2000 Oe) increases with f reaching a saturation value at high f.

Apart from the magneto-dielectric coupling the magneto-electric coupling between the two constituents in the composite has also been investigated. Variation of magneto-electric coupling constant α with respect to d.c. bias magnetic field and at frequencies in the range 1 kHz to 15 kHz was studied at room temperature. At all frequencies, coupling constant α is found to increase on increasing thebias magnetic field till 2000 Oe and then decrease on further increasing the field. The decrease of α at large fields is due to a decrease in piezoelectric coefficient of the relaxor phase SBN50 at large strains created by the magnetostrictive phase CCFO at large bias fields. This trend is shown in Fig. 7(a) for 5 kHz frequency. It is observed that in CoCr_x_Fe_1−x_O_4_, magnetostriction increases up to ~2000 Oe field and then decreases for x = 0.38^30^. Similar behavior ofα in the composite suggests a strong strain mediated magneto-electric coupling in the material. The rate of decrease of magnetostrictive strain in CoCr_0.38_Fe_0.62_O_4_ for fields >2000 Oe however decreases but does not reach saturation for fields as large as 10000 Oe and has a strain of ~25 ppm at this large field. This results in a significant magnetoelectric coupling constant of the composite at 3000 Oe external d.c. bias field, maximum field investigated in the present work and is shown in Fig. 7(a). The variation of α with frequency at a fixed magnetic field of 2000 Oe is shown in Fig. 7(b).The coupling constant α increases on increasing the frequency and attains a value of ~20.6 mVcm^−1^Oe^−1^at 15 kHz frequency in a field of 2000 Oe at room temperature, a relatively high value for a 3-0 bulk composite.

The magnetoelectric coupling in these composites is a product property as it is a combination of magnetostriction leading to dielectric polarization via the piezoelectric property of the ferroelectric. The magnetoelectric response of the composite CCFO/SBN-50 studied in the present work is not exactly similar to the conventional scheme mentioned. The magnetic component CCFO also exhibits dielectric relaxor behavior which is coupled to the dielectric relaxor behavior of SBN50. At room temperature both these components exhibit a dielectric permittivity enhancement due to local polarization relaxor behavior. As a result the net magnetoelectric change in α is due to a combination of inherent behavior of CCFO and the change induced in SBN50 due to the magnetostrictive behavior of CCFO. The magnetostrictive deformation in CCFO induces piezoelectric charge generation in SBN50 together with self-charge generation in both components. The magnetic CCFO phase has an extremely large magnetostrictive strain rate derivative at very low fields which results in non-zero magneto-electric coupling at these low fields in the composite. The self-magnetoelectrive behavior and remnant magnetization in CCFO could also plausibly contribute to α at these low fields. The microstructure, uniform distribution and connectivity of the magnetic component in the relaxor ferroelectric matrix facilitates effective strain transfer and hence realization of relatively large magnetoelectric effect. These results show the feasibility of resonance peak tuning using electric field alone which can produce mechanical deformation in the piezoelectric component. This can in turn induce magnetization change of magnetostrictive phase through inverse magnetoelectric effect which will result in changing the resonance frequency of the composite.

### Composite microwave resonator

**Figure 8:**
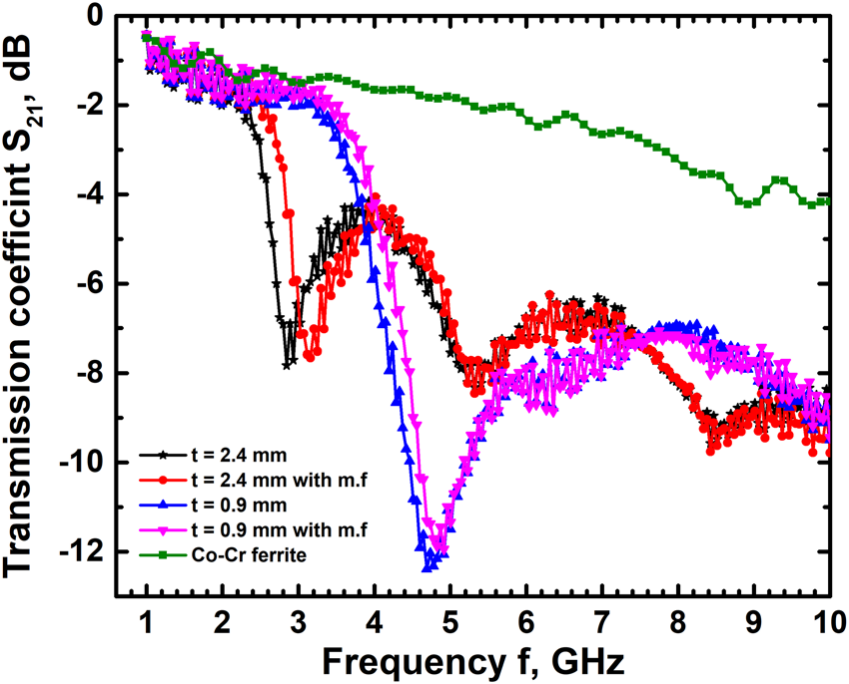
The microwave transmission coefficient S_21_ of the composites show resonance peaks while that of CCFO does not show any absorption peaks in the range 1 GHz to 10 GHz. The resonant frequency peaks of transmission coefficient of the composite shift to higher values on application of an external magnetic field, m.f of 5000 Oe. The 2.4 mm thick disc shows peaks corresponding to 2 orders while the 0.9 mm thick disc shows only first order peak. The transmission coefficient is measured at room temperature.

Communication networks rely heavily on dielectric resonators/filters which carry signals of a specific frequency and also act as filters to remove noise. The resonant frequency of devices made of ferrites in these networks require tuning/switching which is accomplished by applying an external magnetic field. Magnetic tuning however is inherently slow and requires large power compared to electrical tuning. In this context, multiferroic materials wherein the magnetization and electric polarization are coupled offer the flexibility to tune the operating frequency either by applying an electric field or magnetic field. The microwave resonator frequency depends on the effective dielectric permittivity, loss and size of the resonator. To a first approximation, the resonant frequency is given by the relation ${f}_{0}=c/D{[{\varepsilon }_{r}(f)]}^{1/2}\,$where c is the speed of light in vacuum, ε_r_(f) the frequency dependent dielectric permittivity and D the resonator size^47^. The resonant frequency therefore is a non-trivial function of resonator size and its dielectric constant. Changing the size of the resonator does not result in simple inverse variation of f_0_ with D as ε_r_ is not a constant but varies with f, the measuring frequency. Hence the resonant frequency has a unique dependence on D and ε_r_(f). The magneto electric coupling in the composite leads to changes in the effective dielectric permittivity and hence facilitates frequency tuning. Hence in the present work a simple disc shaped microwave resonator/filter was made of both CCFO and SBN50-CCFO composite and tested for frequencies in the range 1–10 GHz. Transmission coefficient S_21_ of CCFO and the composites are shown in Fig. 8. It is observed that CCFO exhibits very small loss which increases nearly linearly with increasing frequency in the range 1 GHz to 10 GHz. This is because the low dielectric permittivity of CCFO leads to microwave resonance at frequencies >10 GHz which have not been probed in the present work. The composite with a thickness of 2.4 mm on the other hand exhibits clear absorption peaks at 2.89 GHz and 5.36 GHz frequency corresponding to first and second order resonance while the composite with a thickness of 0.9 mm exhibits a first order resonance peak at 4.76 GHz. These resonant frequencies shift to higher values on application of an external magnetic field of strength 5000 Oe. In the case of 2.4 mm thick composite the absorption peaks shift to 3.2 GHz and 5.42 GHz respectively while the 0.9 mm thick composite has the peak shifted to 4.87 GHz. These results clearly show that the resonant frequency depends on the effective dielectric constant at any given frequency, size of the filter and magnitude of external magnetic field applied. The shift of resonant frequency in the presence of external magnetic field, independent of the size of filter, indicates tunability and strength of magneto-dielectric coupling in the composite. The transmission loss as well as width of resonant peaks remains nearly constant in both the cases with the quality factor remaining a constant at ~8, independent of external magnetic field. These results clearly show the potential of using this magneto-electric composite as a tunable microwave resonator/filter.

## Conclusions

Single phase materials which show multiferroic behaviour at room temperature are very difficult to come across due to the conflicting nature of properties requirement for the two orders. Hence a composite consisting of magnetostrictiveCoCr_0.4_Fe_1.6_O_4_ and relaxor ferroelectric niobate Ba_0.5_Sr_0.5_Nb_2_O_6_ has been synthesized to investigate the coupling between them. At room temperature the ferrite is known to be magnetostrictive while the niobate is known to exhibit a relaxor like ferroelectric behaviour making them ideal to show multiferroism. It is observed that the magnetostrictive ferrite has a diffuse dielectric phase transition due to ionic hopping close to its magnetic Curie temperature indicating the possibility of coupling between the two orders. The non-magnetic relaxor ferroelectric is found to exhibit two distinct phase transitions due to the presence of multiple ferroelectric phases at room temperature which lead to frustration. At low temperatures the structure develops an incommensurate superlattice whose lattice parameters vary with temperature. The large magnetodielectric as well as magnetoelectric studies show that the two constituents are indeed strongly coupled. A microwave resonator made of this composite exhibits resonance peaks which are sensitive to external magnetic field. This clearly shows not only the presence of strong coupling but also the possibility of realizing electrically tuneable multiferroic microwave resonators for practical application.This aspect however has not been directly investigated in the present work. The resonant frequency is found to be a function of coupled parameters such as effective dielectric constant, size of the resonator as well as magnetic field, which makes it difficult to predict the inter-dependence.

### Data Availability Statement

The datasets generated during and/or analysed during the current study are available from the corresponding author on reasonable request.

## Electronic supplementary material

Supporting Information
