# Supplementary material for: Magnetoelectric and magnetodielectric coupling and microwave resonator characteristics of Ba0.5Sr0.5Nb2O6/CoCr0.4Fe1.6O4 multiferroic composite
Source: Sci Rep. 2018 Aug 2;8:11619. doi: 10.1038/s41598-018-30132-2 (PMC6072765; doi:10.1038/s41598-018-30132-2)
Supplement: Supplementary file 1 — Supporting Information [file 41598_2018_30132_MOESM1_ESM.docx]

**Magnetoelectric and magnetodielectric coupling and microwave resonator characteristics of Ba_0.5_Sr_0.5_Nb_2_O_6_/CoCr_0.4_Fe_1.6_O_4_ multiferroic composite**

**Shivangi Tiwari and Satish Vitta***

**Department of Metallurgical Engineering and Materials Science**

**Indian Institute of Technology Bombay**

**Mumbai 400076; India.**

**Supporting Information**

**Structure:** The room temperature powder x-ray diffraction pattern obtained from Ba_0.5_Sr_0.5_Nb_2_O_6_ is shown in Figure S1(a) together with the results of Rietveld refinement. It can be seen that the compound is in a single phase state with a tetragonal P4bm tungsten bronze structure. The different atomic positions together with the ionic occupancy factors are given in Table S1. The crystal structure generated based on the structural refinement parameters is shown in Figure S1(b). It is seen that the structure is made of NbO_6_ corner shared octahedra which results in the formation of 3 types of interstitial sites with different effective size. The large pentagonal sites are occupied by either Sr^2+^ or Ba^2+^ while the tetragonal sites are occupied only by Sr^2+^. The large Ba^2+^ ion occupies the pentagonal sites with the smaller Sr^2+^ ions occupying only ¼ of the pentagonal sites. It is found that the smaller trigonal sites are totally unoccupied by any of the cations. The powder x-ray diffraction pattern of the ferrite is shown in Figure S2(a) together with the structural refinement results. This compound has the cubic Fd$\bar{3}$m spinel structure and shows formation of a single phase with no impurities. The Co-ferrite compound is known to have an inverse spinel structure with the Fe^3+^ ions having higher probability to occupy the tetrahedral sites compared to Co^2+^ ions. The trivalent Cr^3+^ on the other hand occupies the octahedral sites replacing the Fe^3+^ ions. Hence in the substituted compound CoCr_0.4_Fe_1.6_O_4_ the divalent Co^2+^ ions will occupy the tetrahedral sites together with Fe^3+^ ions while Fe^3+^, Co^2+^ and Cr^3+^ ions should occupy the octahedral sites. Therefore the structural refinement of x-ray diffraction results has been performed using this scheme and it is found that 20 % substitution of Cr^3+^ for Fe^3+^ results in ~ 20 % of Co^2+^ ions being shifted to tetrahedral sites from octahedral sites. The resulting structure therefore can be represented as (Fe_0.8_Co_0.2_)[Fe_0.8_Co_0.8_Cr_0.4_]O_4_. A schematic structure based on these occupancies is shown in Figure S2(b) and the various unit cell parameters are given in Table S2. These two single phase compounds were mixed in the ratio 0.7:0.3 to form the 3-0 magnetoelectric composite and the powder x-ray diffraction pattern obtained from this composite is shown in Figure S3. All the peaks in the diffraction pattern could be indexed to the two single phases, clearly showing that no extra/impurity phases form during sintering of the composite. The fraction of two phases in the composite as determined from x-ray diffraction results was found to be in close agreement with the anticipated value of 0.7:0.3 of ferroelectric and ferromagnetic constituents respectively.

The microstructure of resulting 3-0 composite was studied in the scanning electron microscope to understand the spatial distribution of the two components and the microstructure is shown in Figure S4. The secondary electron image, Figure S4(a) shows the formation of dense structure with no porosity and the density of the pellets was found to be 5.07 gcm^-3^, ~ 95 % of theoretical density of the composite. The secondary electron image however does not delineate the two components in the microstructure. Hence back scattered electron imaging was performed and is shown in Figure S4(b). The two components, ferroelectric and ferrimagnetic can be clearly seen due to their molecular mass contrast and this facilitates determination of grain size of the individual components. The grain size distributions were found to be very different as seen in the two bar graphs, Figure S4(c). The SBN50 grains are large with majority of the grains in the 2 – 8 μm range while the CCFO grains are much smaller with majority of the grains having size < 3 μm. This difference in size distributions leads to an efficient packing with high density and low porosity in the composite. The formation of a dense structure with random distribution of the two phases is extremely important for achieving high coupling between the two phases.

**Table S1:** The atomic positions and lattice parameters obtained from refinement of x-ray diffraction data of the piezoelectric compound Ba_0.5_Sr_0.5_Nb_2_O_6_ are given here. The compound has a tetragonal tungsten bronze crystal structure with the lattice parameters a = 1.2463(9) nm and c = 0.3948(8) nm. The χ^2^ value for the refinement is 2.93.

| **Ba_0.5_Sr_0.5_Nb_2_O_6_** | | | | | |
| --- | --- | --- | --- | --- | --- |
| **Atoms** | **Wyckoff Positions** | **x** | **y** | **z** | **Occupancy** |
| Nb1 | 2 b | 0 | 0.5 | 0.22803 | 1 |
| Nb2 | 8 d | 0.07575 | 0.21038 | 0.21780 | 1 |
| Sr1 | 2 a | 0 | 0 | 0.65264 | 1 |
| Sr2 | 4 c | 0.17276 | 0.67276 | 0.68734 | 0.25 |
| Ba1 | 4 c | 0.17276 | 0.67276 | 0.68734 | 0.75 |
| O1 | 8 d | 0.33984 | 0.01634 | 0.21327 | 1 |
| O2 | 8 d | 0.1429 | 0.06401 | 0.25193 | 1 |
| O3 | 4 c | 0.27517 | 0.77517 | 0.26587 | 1 |
| O4 | 2 b | 0.5 | 0 | 0.67201 | 1 |
| O5 | 8 d | 0.56819 | 0.28918 | 0.65869 | 1 |

**Table S2:** The atomic positions and lattice parameters obtained from refinement of x-ray diffraction data of the magnetostrictive compound CoCr_0.4_Fe_01.6_O_6_ are given here. The compound has a inverse cubic spinel crystal structure with a lattice parameter of 0.8384(2) nm and the χ^2^ value for the refinement is 1.38.

| **CoCr_0.4_Fe_1.6_O_4_** | | | | | |
| --- | --- | --- | --- | --- | --- |
| **Atoms** | **Wyckoff Positions** | **x** | **y** | **Z** | **Occupancy** |
| Fe1 | 8 a | 0.125 | 0.125 | 0.125 | 0.8 |
| Co1 | 8 a | 0.125 | 0.125 | 0.125 | 0.2 |
| Fe2 | 16 d | 0.5 | 0.5 | 0.5 | 0.4 |
| Co2 | 16 d | 0.5 | 0.5 | 0.5 | 0.4 |
| Cr1 | 16 d | 0.5 | 0.5 | 0.5 | 0.2 |
| O1 | 32 e | 0.25(8) | 0.25(8) | 0.25(8) | 1 |





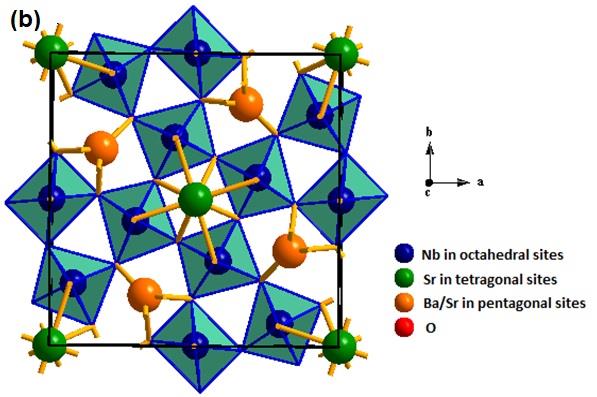


**Figure S1:** The x-ray diffraction pattern obtained from the piezoelectric compound Ba_0.5_Sb_0.5_Nb_2_O_6_ together with the pattern obtained after refinement of structure are shown in (a). The crystal structure and the different atomic positions obtained by refinement are shown in (b).


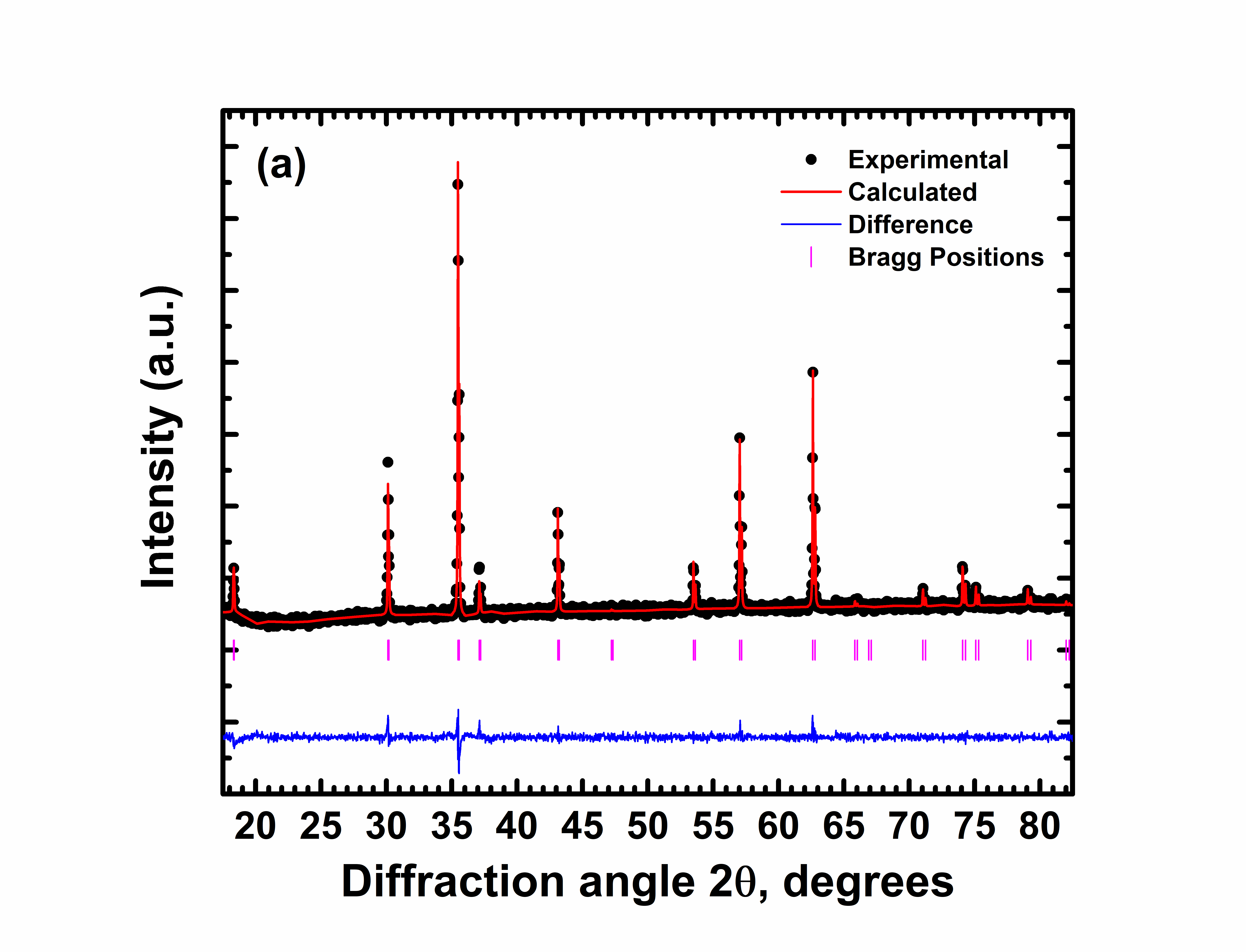

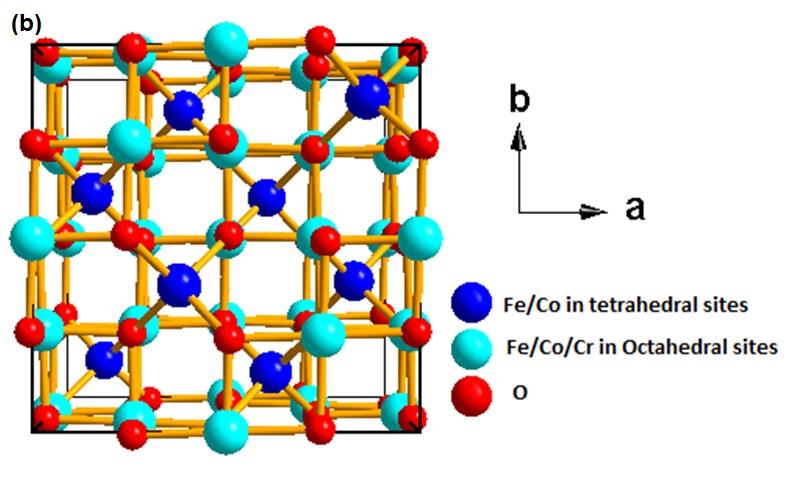


**Figure S2**: The x-ray diffraction pattern together with the pattern obtained by structural refinement for the magnetostrictive ferrite CoCr_0.4_Fe_1.6_O_4_ is shown in (a). The crystal structure obtained after refinement of the x-ray diffraction data is shown in (b) together with the atomic positions.


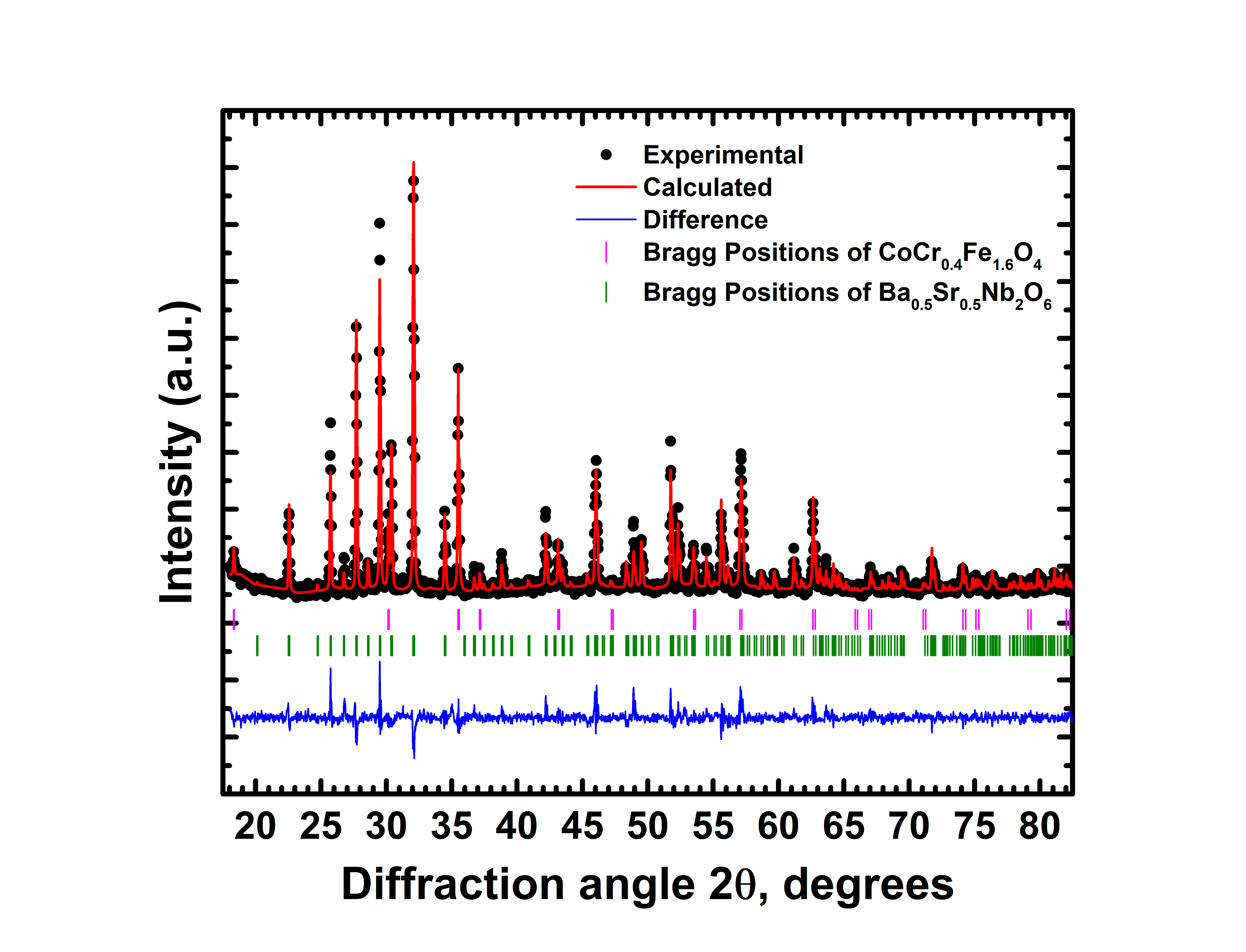


**Figure S3:** The x-ray diffraction pattern obtained from the composite shows peaks corresponding to the two phases - Ba_0.5_Sb_0.5_Nb_2_O_6_ and CoCr_0.4_Fe_1.6_O_4_ with no additional peaks. The diffraction pattern could be simulated as a combination of the two phases. Both the experimental data and the results of refinement are shown here.


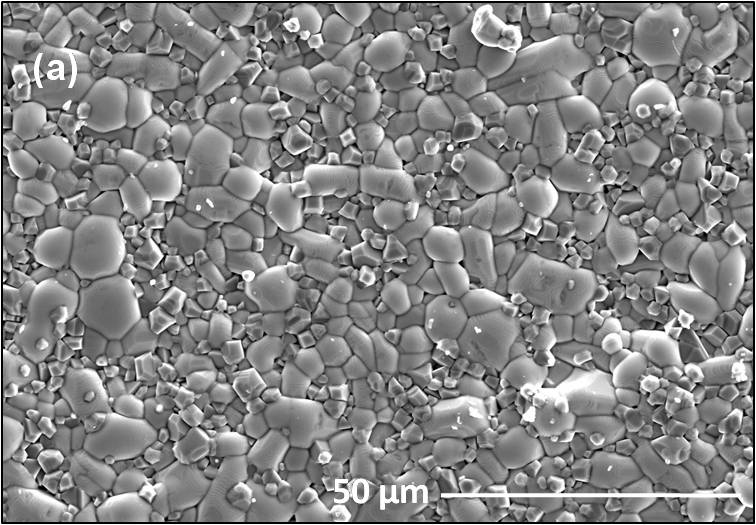

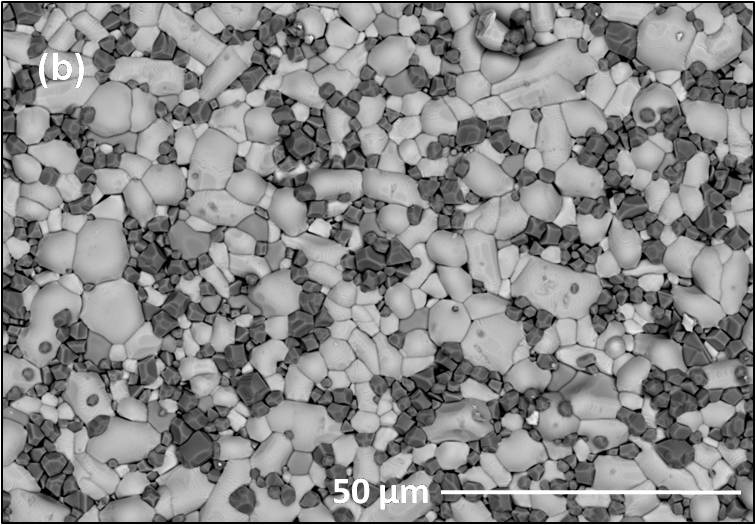

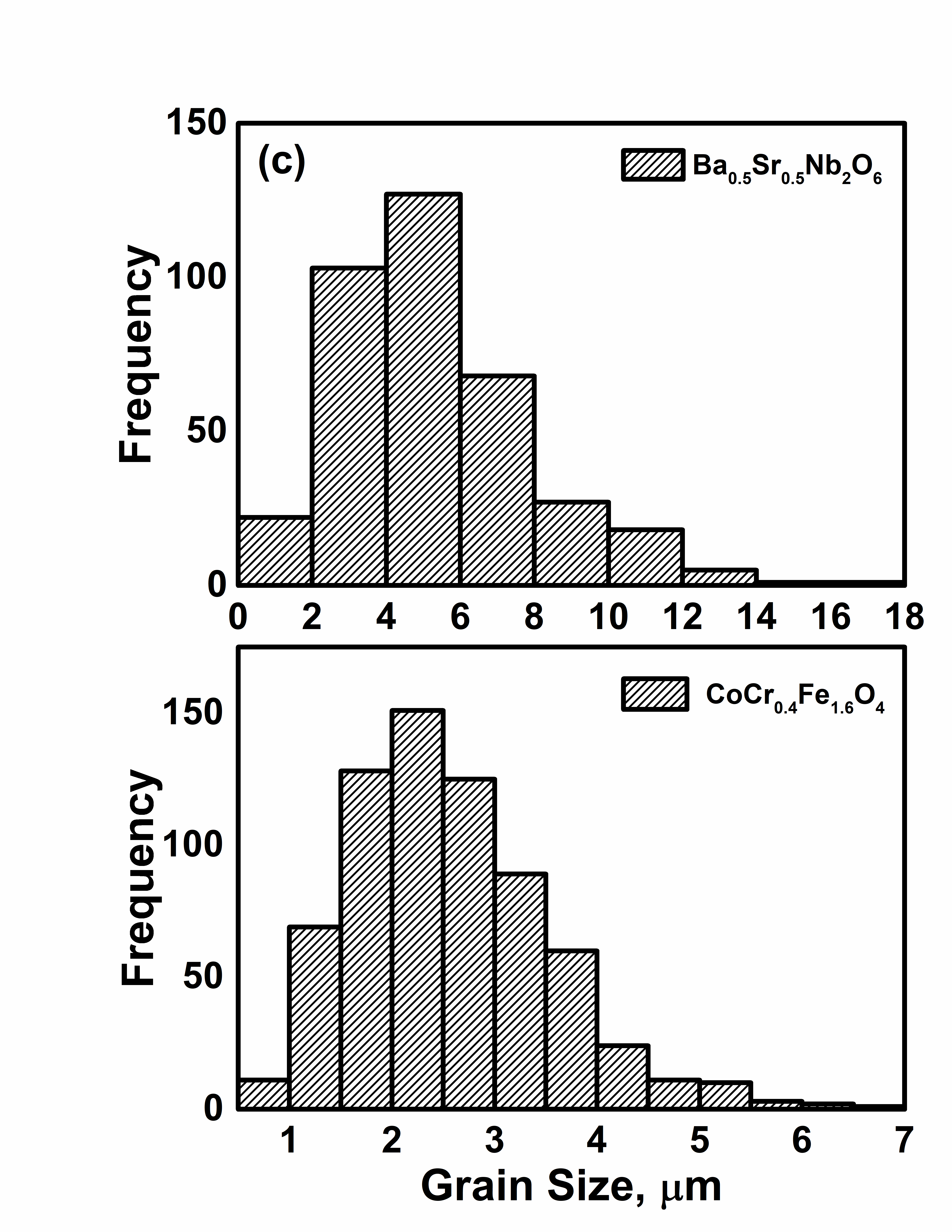


**Figure S4:** The secondary electron image of the composite obtained in a scanning electron microscope (a) shows the presence of a dense microstructure with the two phases randomly distributed and in intimate contact. In order to delineate the two components back scattered electron imaging was also performed and is shown in (b). The black grains of the magnetostrictive component are distributed in the matrix of white grains of the piezoelectric component. The grain size distribution of the two phases is distinctly different as shown in (c) with different average grain sizes. The piezoelectric grains are large compared to the magnetostrictive grains.
